# Explainable Machine Learning Techniques To Predict Amiodarone-Induced Thyroid Dysfunction Risk: Multicenter, Retrospective Study With External Validation

**DOI:** 10.2196/43734

**Published:** 2023-02-07

**Authors:** Ya-Ting Lu, Horng-Jiun Chao, Yi-Chun Chiang, Hsiang-Yin Chen

**Affiliations:** 1 Department of Clinical Pharmacy School of Pharmacy Taipei Medical University Taipei Taiwan; 2 Department of Pharmacy Wan Fang Hospital Taipei Medical University Taipei Taiwan

**Keywords:** amiodarone, thyroid dysfunction, machine learning, oversampling, extreme gradient boosting, adverse effect, resampling, thyroid, predict, risk

## Abstract

**Background:**

Machine learning offers new solutions for predicting life-threatening, unpredictable amiodarone-induced thyroid dysfunction. Traditional regression approaches for adverse-effect prediction without time-series consideration of features have yielded suboptimal predictions. Machine learning algorithms with multiple data sets at different time points may generate better performance in predicting adverse effects.

**Objective:**

We aimed to develop and validate machine learning models for forecasting individualized amiodarone-induced thyroid dysfunction risk and to optimize a machine learning–based risk stratification scheme with a resampling method and readjustment of the clinically derived decision thresholds.

**Methods:**

This study developed machine learning models using multicenter, delinked electronic health records. It included patients receiving amiodarone from January 2013 to December 2017. The training set was composed of data from Taipei Medical University Hospital and Wan Fang Hospital, while data from Taipei Medical University Shuang Ho Hospital were used as the external test set. The study collected stationary features at baseline and dynamic features at the first, second, third, sixth, ninth, 12th, 15th, 18th, and 21st months after amiodarone initiation. We used 16 machine learning models, including extreme gradient boosting, adaptive boosting, k-nearest neighbor, and logistic regression models, along with an original resampling method and 3 other resampling methods, including oversampling with the borderline-synthesized minority oversampling technique, undersampling–edited nearest neighbor, and over- and undersampling hybrid methods. The model performance was compared based on accuracy; Precision, recall, *F*_1_-score, geometric mean, area under the curve of the receiver operating characteristic curve (AUROC), and the area under the precision-recall curve (AUPRC). Feature importance was determined by the best model. The decision threshold was readjusted to identify the best cutoff value and a Kaplan-Meier survival analysis was performed.

**Results:**

The training set contained 4075 patients from Taipei Medical University Hospital and Wan Fang Hospital, of whom 583 (14.3%) developed amiodarone-induced thyroid dysfunction, while the external test set included 2422 patients from Taipei Medical University Shuang Ho Hospital, of whom 275 (11.4%) developed amiodarone-induced thyroid dysfunction. The extreme gradient boosting oversampling machine learning model demonstrated the best predictive outcomes among all 16 models. The accuracy; Precision, recall, *F*_1_-score, G-mean, AUPRC, and AUROC were 0.923, 0.632, 0.756, 0.688, 0.845, 0.751, and 0.934, respectively. After readjusting the cutoff, the best value was 0.627, and the *F*_1_-score reached 0.699. The best threshold was able to classify 286 of 2422 patients (11.8%) as high-risk subjects, among which 275 were true-positive patients in the testing set. A shorter treatment duration; higher levels of thyroid-stimulating hormone and high-density lipoprotein cholesterol; and lower levels of free thyroxin, alkaline phosphatase, and low-density lipoprotein were the most important features.

**Conclusions:**

Machine learning models combined with resampling methods can predict amiodarone-induced thyroid dysfunction and serve as a support tool for individualized risk prediction and clinical decision support.

## Introduction

Amiodarone-induced thyroid dysfunction (AITD) is a common, irreversible, and unpredictable adverse thyroid effect, leading to therapy failure and significant mortality. As it is the drug of choice for arrhythmias and atrial fibrillation, developing predictive models for the early detection of AITD is warranted [1-4]. The incidence of AITD varies with iodine intake and ranges from 17% to 30% [5-7]. Studies indicate that AITD onset is unpredictable [8], and it is followed by significant morbidity and mortality [9]. Amiodarone has a long and variable half-life of approximately 60 to 142 days [10-14], causing difficulty in treating its side effects. Timely and precise patient stratification to identify patients at high risk of AITD is the foremost strategy for preventing life-threatening adverse thyroid effects.

Predicting AITD requires advanced data-mining skills to unveil its multifactorial mechanisms. Older age, female sex, chronic obstructive pulmonary disease, chronic kidney disease, and underlying autoimmune thyroid disorders contribute to AITD [6,15-22]. Previously, statistical approaches were used to develop a risk prediction index for AITD for adults with congenital heart disease [23] and to perform an AITD risk factor analysis [24-26]. However, these studies failed to capture dynamic factors, as they collected data at a single time point. A limited sample size, heterogeneous patient cohort, single data-collection time point, and lack of consideration of factorial interactions further contributed to suboptimal predictive performance. In another report, a machine learning algorithm was used to study immune checkpoint inhibitor–induced thyroid dysfunction and was found to have an area under the receiver operating characteristic curve (AUROC) of 0.77 [27]. This model outperformed conventional regression models for predicting multiple diseases, such as hypertension [28], neck pain [29], and hepatocellular carcinoma [30]. The robust nature of machine learning techniques could be promising for building a surveillance system for AITD in comparison to traditional regression methods.

Combining machine learning and resampling strategies can counteract imbalanced data resulting from the low incidence of AITD in the real world. Tree-based ensemble learning methods, such as extreme gradient boosting (XGBoost) and adaptive boosting (AdaBoost), are commonly applied for imbalance classification [31,32]. K-nearest neighbors (KNN) with data resampling methods perform well in imbalance classification [33]. Combined with the synthetic minority oversampling technique (SMOTE), these methods rebalance the minority and achieve promising performance in disease and survival prediction [34,35]. Borderline SMOTE further improves internal data distribution by using samples on the boundary to synthesize new instances and is able to diagnose lung cancer early [36]. Edited nearest neighbor (ENN) removes ambiguous data from the majority class, while borderline synthetic minority oversampling technique–edited nearest neighbor (B-SMT-ENN) is a hybrid technique that performs oversampling by SMOTE and undersampling by ENN. These hybrid sampling methods successfully improve adverse-effect predictions and have other medical applications [37-39].

An accurate machine learning prediction model with a resampling strategy can be applied to overcome imbalanced data for multifactorial adverse effects. The objectives of this study were to develop and validate machine learning models for forecasting individualized AITD risk with imbalanced real-world data. The performance of 12 models with 4 machine learning classifiers, including XGBoost, AdaBoost, KNN, and logistic regression (LR), along with 3 resampling methods (borderline-SMOTE, ENN, and B-SMT-ENN), were compared. The specific aims of this study were (1) to select a fine-tuned model for AITD prediction with multiple performance metrics, including accuracy; Precision, recall, *F*_1_-score, G-mean, AUROC, and AUPRC, and (2) to optimize machine learning-based risk stratification schemes for AITD by readjusting the decision thresholds for individualized risk prediction.

## Methods

### Ethical Considerations

This retrospective study used a delinked clinical research database from 3 hospitals in the Taipei Medical University health care system, including Taipei Medical University Hospital, Wan Fang Hospital, and Shuang Ho Hospital. The study was approved by the Taipei Medical University Joint Institutional Review Board (N202107054). As the data were deidentified, the requirement for informed consent was waived. This study adhered to the TRIPOD (Transparent Reporting of a Multivariate Prediction Model for Individual Prognosis or Diagnosis) checklist [40].

### Study Design

**Figure 1:**
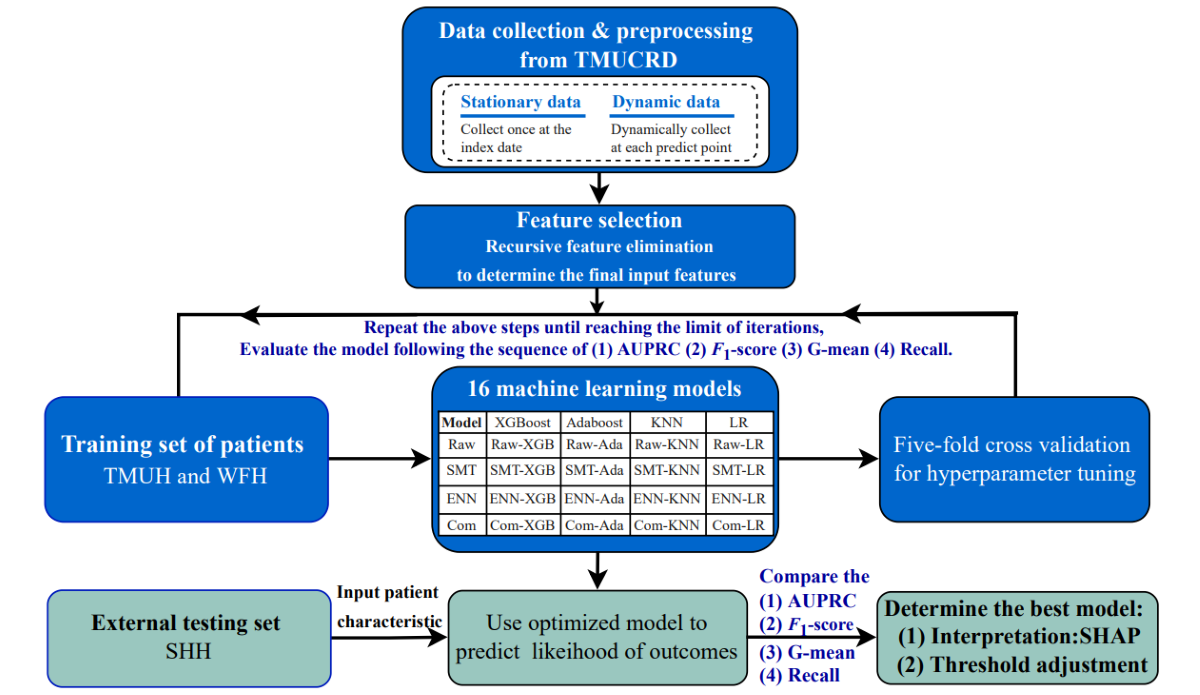
Study design to construct machine learning models for predicting amiodarone-induced thyroid dysfunction. The test set performance was evaluated in the same sequence as the training set. The green boxes indicate that the validation process was completed. Ada: adaptive boosting; AUPRC: area under the precision-recall curve; B-SMT: oversampling with borderline synthetic minority oversampling technique (borderline SMOTE); ENN: edited nearest neighbor (ENN); G-mean: geometric mean; Hyb: hybrid oversampling with borderline synthetic minority oversampling technique and undersampling with edited nearest neighbor (B-SMT-ENN); KNN: k-nearest neighbor; LR: logistic regression; SHAP: Shapley additive explanations; SHH: Shuang Ho Hospital; SMT: synthetic minority oversampling technique; TMUCRD: Taipei Medical University clinical research database; TMUH: Taipei Medical University Hospital; WFH: Wan Fang Hospital; XGB: extreme gradient boosting.

Figure 1 shows the study design, including data collection, feature selection, model construction, and 5-fold internal validation using a training set, external validation by a test set, and model interpretation. The training set of the study comprised patients from Wan Fang Hospital and Taipei Medical University Hospital, whereas the external test set comprised patients from Shuang Ho Hospital. There were 16 machine learning models built by the training set. Model performance was compared with the external testing set.

### Patient Cohort

Patients older than 18 years who had first been prescribed oral amiodarone between January 2005 and December 2017 were included. Patients were excluded if they were pregnant or had a history of a thyroid disorder diagnosis, thyroid surgery, or subclinical thyroid laboratory data (Multimedia Appendix 1) within 1 year before the index day, which was the day of the first amiodarone prescription. The predefined inclusion and exclusion criteria were modified from previous medication-induced thyroid dysfunction research [6,27]. The study subjects were followed up for 2 years after the index date. This study collected features until the end of the study period and recorded patient loss to follow-up and the occurrence of AITD events.

### Data Collection and Preprocessing

The study collected stationary and dynamic features from the clinical research database. Stationary features, including sex, age, and BMI, were collected once at the index date. Dynamic features, including laboratory tests, comorbidities, and comedications, were continuously collected to reflect the patient’s clinical condition. The collection points for dynamic features were at baseline and the first, second, third, sixth, ninth, 12th, 15th, 18th, and 21st months after amiodarone initiation. Details of the dynamic data collection are shown as a diagram in Multimedia Appendix 2. The features reengineered by the research team included the accumulated or average dose of amiodarone and annual time-series variations in laboratory tests.

Robust scaler algorithms were used to normalize the data and reduce the effect of extreme numeric variables and large differences in range between laboratory values [41]. Variables with more than 90% missing data were deleted from the machine learning programs. Missing values were first imputed with the last observation carried forward, and then the remainders were imputed with Multivariate Imputation by Chained Equations (Scikit-learn), an open-source imputation software package [42-44]. Zero was imputed for laboratory data with changing rates, such as the trend or slope of lab values. The codebook and missing rates in the training and test sets are provided in Multimedia Appendix 3.

### Dynamic Prediction of the Study Outcome

The outcome for prediction in this study was the occurrence of AITD, which was defined by a thyroid function test and with diagnostic criteria from previous studies [6,22,26,45]. Cases of AITD were identified if the thyroid-stimulating hormone (TSH) titer was <0.1 mU/l and the free thyroxin (fT4) level was higher than the normal range, while cases of amiodarone-induced hypothyroidism were ascertained by a serum TSH titer of >10 mU/l, regardless of the fT4 level; a TSH titer of 4 to 10 mU/l with a lower than normal fT4 level; an International Classification of Diseases (ICD)-9 code 242, 243, or 244 or an ICD-10 code E02, E03, E05, or E06; having received pharmacotherapy (eg, levothyroxine; Propylthiouracil, carbimazole, or methimazole) for thyroid disease; or an ICD-9 procedure code for a thyroidectomy. As TSH and fT4 are definite diagnostic criteria for AITD, the last data values before the prediction point were masked to avoid data leakage in the test set. Once a patient developed AITD, the data at that time point were coded 1, and it was designated the earliest AITD onset date.

### Model Construction With the Training Data Set

Hyperparameter tuning of the XGBoost, AdaBoost, KNN, and LR algorithms was performed with an exhausted-grid search toward maximizing *F*_1_-score metrics. Five-fold cross-validation was performed inside each grid option, and the optimal hyperparameter set was chosen based on the model in the grid search with the highest *F*_1_-score. XGBoost was tuned on 7 hyperparameters, including *max_depth*, *min_child_weight*, *gamma*, *subsample*, *colsample_bytree*, *n_estimators*, and *learning_rate* for 94,080 grid options. Three hyperparameters of AdaBoost that were tuned included *n_estimators*, *learning rate*, and *algorithm*, with 160 combinations. As the KNN is based on the KNN of the prediction point, *n_neighbors*, *weights*, and *metric*, combining 120 sets of parameters, were tested. Finally, the study used an LR established with the *scikit-learn* module for binary outcome classification. *P Penalty*, *solvers*, and *C*, hyperparameters of LR, were calculated in 140 selections. Details of the hyperparameters and the final best combination of the above 16 models are shown in Multimedia Appendix 4.

Recursive feature elimination (RFE) with cross-validation was used for the training set. Pseudocodes of the grid search and cross-validation are presented in Multimedia Appendix 5. The minimal number of feature sets was generated by XGBoost and AdaBoost. As the incidence of AITD in the training and test sets was 14.3% and 11.4%, respectively, the imbalance issue was managed by (1) oversampling with the borderline synthetic minority oversampling technique (B-SMT) [46]; (2) undersampling the majority class with ENN [47]; and (3) a combination of oversampling and undersampling with B-SMT-ENN. The raw strategy and 3 resampling strategies were applied to XGBoost, AdaBoost, KNN, and LR, as shown in Figure 1. This study finally constructed 16 models through the 5-fold cross-validation process of the training sets.

### Model Performance Comparison by Test Set

The performance of the 16 models generated with the training data set were validated and evaluated on the test data set. Model performance was compared using accuracy; Precision, recall, *F*_1_-score, geometric mean, AUROC, and AUPRC [48]. The AUPRC, G-mean, and *F*_1_-score were major metrics over AUROC due to the imbalanced data in this study [49,50]. The study also prioritized recall over precision as the major performance index, to minimize the cost of failing to detect AITD [51], while the accuracy; Precision, and AUROC were minor indices. The formulas of each evaluation metric are provided in Multimedia Appendix 6.

### Feature Importance, Threshold Adjustment, and Kaplan-Meier Analysis

This study further analyzed individualized feature importance and survival curves of different thresholds to assess risk factors and differentiate high-risk patients. The Shapley additive explanations (SHAP) python package was used to understand the importance and influence of each risk factor that caused AITD [52]. The contribution of each feature was computed and plotted to interpret the model. The precision-recall (PR) curve of the best model was plotted to determine the optimal cutoff based on the maximum *F*_1_-score. A threshold-moving system was further used by placing different cutoff points on the PR curve for binary classification. Five cutoff points were selected for analysis, including the points to forecast the top 1%, 5%, 15%, and 25% of patients with AITD risk, as well as the one determined by the threshold with the maximized *F*_1_-score [53]. The recall and sensitivity for the above 5 cutoff points were then calculated and compared. A Kaplan-Meier (KM) survival curve was plotted using different cutoff thresholds to compare the actual survival of high- and low-risk groups for statistical comparison.

### Statistical Analysis

Baseline characteristics were evaluated with the chi-square test or Fisher exact test for categorical variables, and independent 2-tailed *t* tests were used for continuous variables. The Wilcoxon rank-sum test was used when the data were not normally distributed. The cumulative thyroid dysfunction incidence was compared with the log-rank test. Data were analyzed using SAS (version 9.4; SAS Institute); Python (version 3.9.5; Python Software Foundation), and R studio (version 1.3.1093; R Studio). The statistical significance of the AUPRC was calculated using MedCalc (MedCalc Software).

## Results

### Baseline Characteristics

The study included 6497 amiodarone users. The results of a univariate analysis of their demographics and other features are shown in Table 1. The Strengthening The Reporting of Observational Studies in Epidemiology (STROBE) flowchart for patient selection is presented in Multimedia Appendix 7. The training set contained 4075 subjects, among whom 583 (14.3%) developed AITD, while the test set had 2422 patients, among whom 275 (11.35%) had AITD. The distribution of gender and mean age did not significantly differ between the training and test sets. The AITD group had a higher proportion of female patients, and its median age was older than that of the non-AITD group.

**Table 1:** Patient demographics and univariate analysis.

Characteristics		Training set (N=4075)				Test set (N=2422)		
		AITD^a^ (n=583, 14.3%)	Non-AITD (n=3492)	*P* value	AITD (n=275, 11.4%)		Non-AITD (n=2147)	*P* value
**Patient demographics**								
	Male, n (%)	282 (48.37)	1969 (56.38)	<.001	121 (44)		1214 (56.54)	<.001
	Age (years), median (IQR)	76.00 (66.00-83.00)	73.00 (62.00-83.00)	.001	75.00 (64.00-83.00)		72.00 (61.00-81.00)	.006
	BMI (kg/m^2^), median (IQR)	24.14 (21.83-26.67)	24.27 (22.04-26.50)	.75	24.94 (22.83-27.04)		24.49 (22.22-26.51)	.02
	Charlson comorbidity index, median (IQR)	1.00 (1.00-2.00)	1.00 (1.00-2.00)	.79	1.00 (0.00-1.00)		1.00 (1.00-2.00)	.003
	Smoking habit, n (%)	11 (1.89)	56 (1.6)	.75	0 (0)		17 (0.79)	.27
	Alcohol habit, n (%)	11 (1.89)	63 (1.8)	>.99	3 (1.09)		38 (1.77)	.57
**Indication for amiodarone, n (%)**								
	Atrial fibrillation	360 (61.75)	223 (38.25)	<.001	178 (64.73)		1192 (55.52)	.004
	Supraventricular tachycardia	95 (16.3)	509 (14.58)	.29	28 (10.18)		321 (14.95)	.04
**Use of amiodarone, median (IQR)**								
	Cumulative dose (g)	18.20 (5.60-39.00)	16.80 (5.00-48.60)	0.49	15.60 (5.60-35.20)		11.60 (3.00-35.00)	.08
	Duration (days)	126.00 (28.00-332.00)	115.00 (25.00-554.00)	.02	125.00 (28.00-290.00)		75.00 (15.00-442.00)	.64
	Prescription daily dose (mg/day)	200.00 (134.09-217.02)	200.00 (136.36-261.91)	.04	200.00 (127.88-216.13)		200.00 (141.61-238.89)	.16
	Average dose per kg body weight (g/kg)	0.30 (0.09-0.62)	0.27 (0.08-0.75)	.64	0.24 (0.08-0.55)		0.18 (0.05-0.55)	.08
**Laboratory data, median (IQR)**								
	Thyroid-stimulating hormone (mU/l)	3.20 (2.79-3.51)	2.08 (1.40-2.50)	<.001	3.22 (2.97-3.40)		2.13 (1.45-2.50)	<.001
	Free thyroxine (ng/dl)	1.18 (1.11-1.25)	1.27 (1.17-1.39)	<.001	1.16 (1.10-1.21)		1.22 (1.10-1.31)	<.001
	High-density lipoprotein cholesterol (mg/dL)	55.03 (47.93-60.39)	53.00 (45.49-59.99)	.001	53.00 (44.00-58.33)		50.64 (40.00-57.32)	.16
	Low-density lipoprotein cholesterol (mg/dL)	89.29 (79.00-101.49)	91.72 (80.45-106.31)	.001	90.00 (81.00-101.00)		92.22 (81.00-110.00)	.12
	Red blood cells (10^6^ cells/µL)	4.21 (3.57-4.42)	4.23 (3.60-4.48)	.06	4.25 (3.66-4.47)		4.24 (3.66-4.51)	.67
	Hemoglobin (g/L)	12.80 (11.00-13.40)	12.87 (10.90-13.60)	.05	12.80 (11.20-13.50)		12.90 (11.10-13.70)	.31
	Hematocrit (%)	37.9 (32.7-39.6)	38.3 (32.7-40.21)	.007	38.18 (33.2-40)		38.35 (33.2-40.6)	.35
	Alkaline phosphatase (U/L)	64.68 (53.66-83.07)	75.85 (60.29-96.00)	<.001	58.70 (47.26-78.10)		75.42 (58.92-95.38)	<.001
	Triglycerides (mg/dl)	86.00 (72.60-119.00)	91 (74.00-124.00)	.02	84.60 (73.86-118.00)		92.00 (76.00-132.00)	.02
**Concurrent medication, n (%)**								
	Allopurinol	30 (5.14)	110 (3.15)	.02	9 (3.27)		88 (4.1)	.62
	Tyrosine kinase inhibitors	1 (0.17)	38 (1.08)	.04	1 (0.36)		9 (0.41)	>.99
	Nonsteroidal anti-inflammatory drugs	218 (37.39)	1135 (32.5)	.02	90 (32.73)		814 (37.91)	.11
	Diabetes mellitus medications	133 (22.81)	638 (18.27)	.01	47 (17.09)		393 (18.3)	.68
	Metformin	94 (16.12)	450 (12.89)	.04	29 (10.54)		287 (13.37)	.22
**Concurrent diseases, n (%)**								
	Hypertension	415 (71.18)	2275 (65.14)	.005	171 (62.18)		1278 (59.52)	.44
	Bradycardia	22 (3.77)	68 (1.94)	.009	6 (2.18)		23 (1.07)	.19
	Diabetes	210 (36.02)	1068 (30.58)	.01	77 (28)		698 (32.51)	.15
	Anemia	94 (16.12)	435 (12.46)	.02	26 (9.45)		254 (11.83)	.29
	Gout	79 (13.55)	338 (9.67)	.005	20 (7.27)		157 (7.31)	>.99
	Chronic renal failure	113 (19.3)	548 (15.69)	.03	32 (11.63)		292 (13.6)	.42
	Renal dysfunction	174 (29.83)	786 (22.51)	<.001	54 (19.64)		459 (21.38)	.56

^a^AITD: amiodarone-induced thyroid dysfunction.

### Model Construction and Evaluation

Feature selection by RFE with 5-fold cross-validation generated 19 features with an accuracy of 0.895 with AdaBoost, while 46 features with an accuracy of 0.914 were generated by XGBoost. Considering the simplicity and accuracy of the model, the 19 features selected by AdaBoost were used for further model development. A figure showing RFE and the features selected is provided in Multimedia Appendix 8.

**Figure 2:**
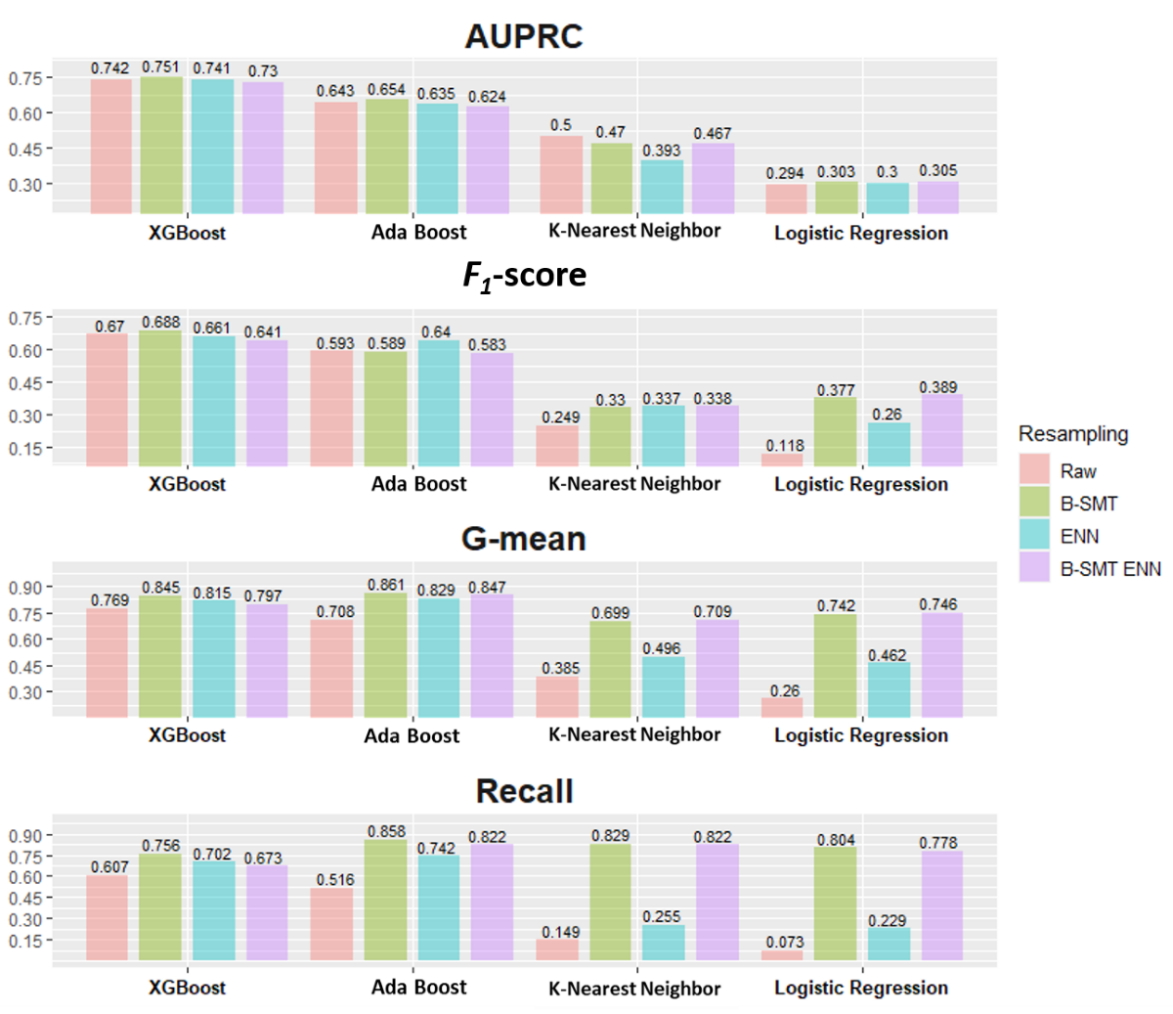
Performance metrics for evaluating a model on imbalanced data. AdaBoost: adaptive boosting; AUPRC: area under the precision-recall curve; B-SMT: borderline synthesized minority oversampling technique; B-SMT ENN: hybrid oversampling with borderline synthetic minority oversampling technique and undersampling with edited nearest neighbor; ENN: edited nearest neighbor.

Figure 2 compares the major model performance indices for the test set: AUPRC, *F*_1_-score, G-mean, and recall. The internal validation performance of the training set is provided in Multimedia Appendix 9. The 4 major performance metrics for the XGBoost and AdaBoost models were consistently higher than for the KNN and LR models, with higher AUPRC and *F*_1_-scores for the XGBoost-based model. Among different resampling methods, the best AUPRC was for XGBoost-B-SMT (0.751, 95% CI 0.697-0.799), which was significantly higher than for XGBoost-Raw (0.742, 95% CI 0.687-0.790; *P*<.05), XGBoost-ENN (0.741, 95% CI 0.686-0.790; *P*<.05), and XGBoost-B-SMT-ENN (0.730, 95% CI 0.675-0.779; *P*<.05). Multimedia Appendix 10 summarizes the results of the statistical comparisons for AUPRCs.

XGBoost-based models also produced higher G-means, with values of 0.688, 0.661, and 0.641 for XGBoost-B-SMT, XGBoost-ENN, and XGBoost-B-SMT-ENN, respectively. The 3 resampling methods all increased G-mean performance for XGBoost and AdaBoost but did not consistently increase the G-mean for KNN or LR. Finally, the recall levels of XGBoost-B-SMT and AdaBoost-B-SMT reached 0.756 and 0.858, respectively, while KNN-ENN only rounded to 0.255.

Table 2 further lists the performance on all metrics, including the minor indices of accuracy; Precision, and AUROC, for the test sets. All models had an AUROC >0.8, except the LR model without resampling. Like the major metrics, the AdaBoost- and XGBoost-based models had higher accuracy and AUROC values compared to the KNN- and LR-based models. The AUROC values for the XGBoost- and AdaBoost-based models were >0.9, while only the accuracy of the XGBoost-based models exceeded 0.9.

**Table 2:** Model performance to predict amiodarone-induced thyroid dysfunction.

Models		Major indices					Minor indices		
		AUPRC^a^	Recall	*F*_1_-score	G-mean^b^	Accuracy		Precision	AUROC^c^
**XGBoost^d^**									
	Raw	0.742	0.607	0.670	0.769	0.932		0.748	0.936
	B-SMT^e^	0.751	0.756	0.688	0.845	0.923		0.632	0.934
	ENN^f^	0.741	0.702	0.661	0.815	0.918		0.624	0.939
	B-SMT ENN^g^	0.730	0.673	0.641	0.797	0.914		0.611	0.924
**AdaBoost^h^**									
	Raw	0.643	0.516	0.593	0.708	0.919		0.696	0.923
	B-SMT	0.654	0.858	0.589	0.861	0.864		0.448	0.921
	ENN	0.635	0.742	0.640	0.829	0.905		0.562	0.922
	B-SMT ENN	0.624	0.822	0.583	0.847	0.867		0.452	0.914
**K-nearest neighbor**									
	Raw	0.500	0.149	0.249	0.385	0.898		0.759	0.835
	B-SMT	0.470	0.829	0.330	0.699	0.617		0.206	0.816
	ENN	0.393	0.255	0.337	0.496	0.886		0.496	0.825
	B-SMT ENN	0.467	0.822	0.338	0.709	0.635		0.213	0.813
**Logistic regression**									
	Raw	0.294	0.073	0.118	0.26	0.877		0.313	0.798
	B-SMT	0.303	0.804	0.377	0.742	0.698		0.246	0.806
	ENN	0.300	0.229	0.260	0.462	0.852		0.300	0.811
	B-SMT ENN	0.305	0.778	0.389	0.746	0.722		0.259	0.803

^a^AUPRC: area under the precision-recall curve.

^b^G-mean: geometric mean.

^c^AUROC: area under the receiver operating characteristic curve.

^d^XGBoost: extreme gradient boosting.

^e^B-SMT: borderline synthesized minority oversampling technique.

^f^ENN: edited nearest neighbors.

^g^B-SMT ENN: hybrid oversampling with borderline synthetic minority oversampling technique and undersampling with edited nearest neighbor.

^h^AdaBoost: adaptive boosting.

### Feature Importance, Threshold Adjustment, and KM Survival Analysis

**Figure 3:**
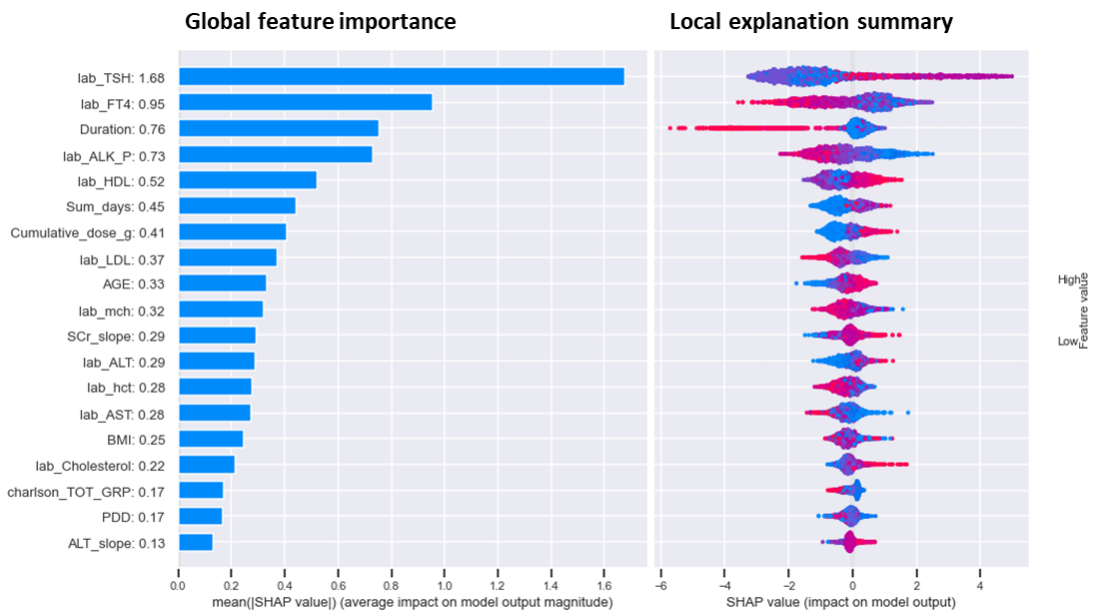
Shapley additive explanations importance plot of the extreme gradient boosting–borderline synthetic minority oversampling technique model. SHAP: Shapley additive explanations.

Figure 3 shows the SHAP summary plot. As shown in this graph, the TSH level had the highest contribution to AITD risk, with a SHAP value of 1.68. The fT4 level, amiodarone treatment duration, alkaline phosphatase level, high-density lipoprotein (HDL) level, and low-density lipoprotein (LDL) level were associated with a higher predicted probability of AITD, with respective SHAP values of 0.95, 0.76, 0.73, 0.52 and 0.37. Furthermore, therapeutic days, cumulative dose, age, and BMI, with SHAP values of 0.45, 0.41, 0.33, and 0.25, respectively, were important global predictors. The local explanation summary plot demonstrated the direction of relationships between clinical variables and AITD, with positive SHAP values indicating higher AITD risk. A higher TSH level was the most informative feature in determining AITD, with a lower fT4 level, shorter treatment duration, lower alkaline phosphatase level, higher HDL level, and lower LDL level increasing the AITD risk. In addition, longer therapeutic days, higher cumulative dose, older age, and lower BMI also raised the AITD risk.

**Figure 4:**
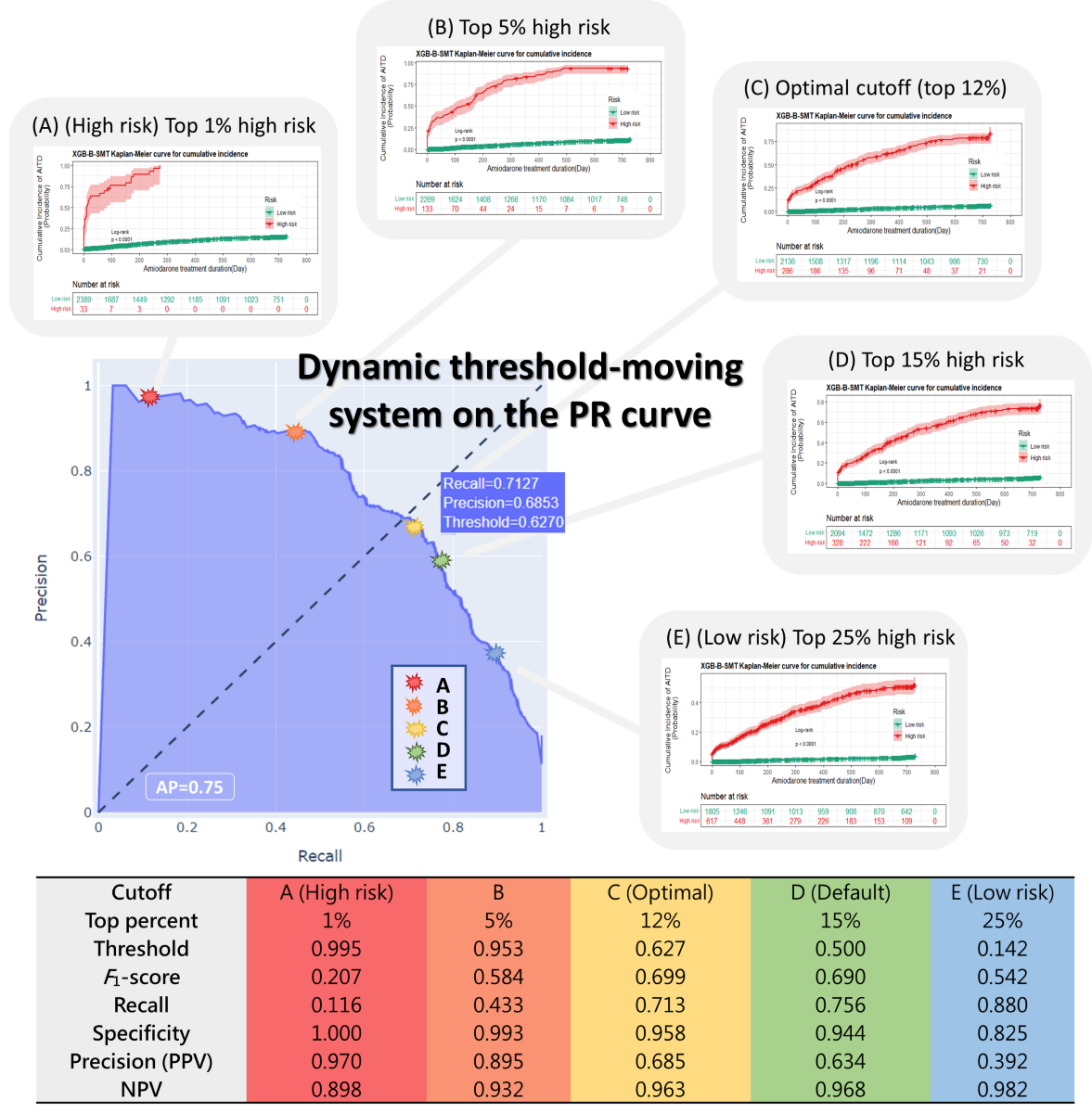
Dynamic and interactive threshold-moving system. The figure represents the contribution of the corresponding features to amiodarone-induced thyroid dysfunction risk. Global feature importance refers to a single ranking of all features for the model, while the local explanation calculated Shapley additive explanation values for each prediction to understand features that contributed to that single prediction. The Kaplan-Meier plot of each threshold is shown in the figure. The yellow star represents the optimal cutoffs for threshold, *F*_1_-score, recall, and precision: 0.627, 0.699, 0.713, and 0.685, respectively. AP: average precision; NPV: negative predictive value; PPV: positive predictive value; PR: precision-recall.

Figure 4 shows KM curves based on using different cutoff points on the PR curve for the XGBoost–borderline SMOTE model. The thresholds of the 5 cutoff points were 0.995 for the top 1% (point A), 0.953 for the top 5% high-risk patients (point B), 0.627 for the optimal point determined by the maximized *F*_1_-score (point C), 0.5 for the top 15% as the default value (point D), and 0.142 for the top 25% (point E) of patients predicted to be at risk of AITD. Moving from default point D, with a threshold of 0.5, to point A, with a threshold of 0.995, the recall significantly decreased from 0.756 to 0.116. When changing the threshold from 0.5 (default; Point D) to 0.142 (point E), recall increased from 0.756 to 0.88. The optimal cutoff (point C), with a threshold of 0.627, yielded better performance, with an accuracy of 0.93 and a precision of 0.685, and the best *F*_1_-score was achieved at 0.699. The corresponding KM curves for the 5 cutoff points were able to differentiate high- and low-risk patients in the log-rank test (*P*<.001). Point A had only 33 within 2422 patients (1.4%) in the high-risk group, and point E predicted 617 within 2422 patients (25.4%) at high risk of AITD. Point C, with 286 high-risk patients within 2422 patients (11.8%), demonstrated an optimal prediction of 275 true positives for patients with AITD.

### Prediction Distribution

**Figure 5:**
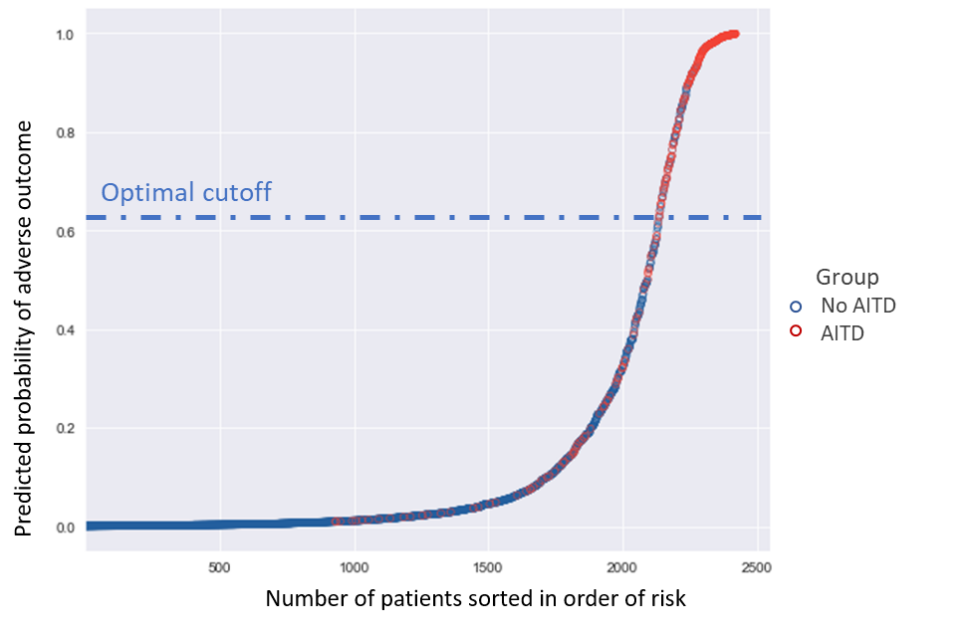
Prediction distribution of adverse thyroid effects in amiodarone users. Patients were sorted in order of risk; red dots represent the AITD group, while blue dots represent the non-AITD group. AITD: amiodarone-induced thyroid dysfunction.

Figure 5 visualizes the prediction distribution of AITD for the 2422 subjects in the external test set. A color change from blue to red indicates increased predicted risk, with a dramatic change occurring around the cutoff point of 0.627. Among 275 patients who developed AITD, 196 (71.3%) had a predicted risk above 0.627 and were determined to be at high risk of AITD according to the XGBoost-borderline SMOTE model. Among the remaining 2147 non-AITD patients, 2057 (95.8%) had a predicted risk lower than the optimal threshold and were thus true negatives estimated by the model.

## Discussion

### Main Findings

This study constructed an explainable and threshold-modifiable machine learning model with the resampling method for AITD risk stratification using dynamic clinical features from the Taipei Medical University clinical research database. The model with the best prediction performance, XGBoost-borderline SMOTE, was validated using external data from another hospital to ensure the credibility and generalizability of the results. It remained robust under conditions of different physicians; Prescription patterns, and hospitals. Resampling methods effectively tackled the imbalanced data and enhanced the model performance. There were 19 clinical features selected by the RFE. Time-series input for dynamic clinical features allowed for real-time assessment and prediction according to a patient’s changing disease state. The SHAP plot provided a better visualization tool to understand the contributions of features to AITD. Modifying the threshold on the PR curve by comparison to the KM curve could improve the help provided for clinical decision-making by determining the percentage of the AITD risk population in different practice settings.

### Best-Performing Model

The outstanding performance of XGBoost-borderline SMOTE in this study resulted from ensemble learning boosting algorithms and a resampling-oversampling technique. As a tree-based ensemble learning algorithm, XGBoost has been shown to be able to detect the complex and potentially nonlinear relationships in imbalance classification, such as in predicting adverse outcomes of chronic heart failure or the adverse effects of analgesics for osteoarthritis [37,54]. In contrast, KNN was sensitive to the majority of instances and thus performed poorly for imbalance classification [55], while the traditional LR model led to biased parameter estimates and classification performance and was less suitable for handling imbalanced data [55-57]. Interestingly, the study also found that oversampling B-SMT consistently outperformed undersampling ENN and hybrid sampling B-SMT-ENN on AUPRC, recall, and G-mean. The undersampling ENN and hybrid sampling methods used a process that deletes samples from the majority class; therefore, valuable information determining the decision boundary between the classes might have been lost. Borderline SMOTE created synthetic data only along the decision boundary, which was the best-performing strategy in this study; it has previously been used to successfully predict chronic kidney disease and hepatitis B virus infection with class imbalance [58,59].

### Feature Identification and Interpretation

This study used SHAP for model interpretation, which allowed precise ranking of variables with clinical reasoning and justification. Top features, such as a high TSH level, short treatment duration, low FT4 level, advanced age, and a higher cumulative amiodarone dose, have been well explained by previous epidemiological studies [6,7,16]. Low alkaline phosphatase and high alanine aminotransferase levels were also found to be associated with hypothyroidism [60]. However, HDL and LDL, which were selected by RFE in this research, were not statistically significantly correlated with thyroid function in previous pharmacoepidemiological reports [61]. Their contributions might be masked by factorial interactions in statistical approaches. Similar phenomena were reported for thyroid disease, chronic kidney disease, and analgesic adverse-effect prediction [54,62,63]. The machine learning models identified relevant features with nonlinear relationships and complex interactions between factors and outcomes, such as HDL and LDL, in the present study; Promising to help doctors and pharmacists to pay special attention when checking amiodarone users’ lipid panels to prevent adverse thyroid events.

### Threshold-Moving System on the PR Curve

This study used a moving threshold system on the PR curve to select optimal cutoff points for assessments with the KM curve. This innovative approach not only allowed comparisons of model performance at different decision thresholds, but also further ensured the capability of the model to differentiate clinically significant high- and low-risk groups. As decision threshold adjustment is a known strategy to deal with imbalanced classification, the best cutoff was based on the maximum *F*_1_-score, as in a previous study of diabetes risk prediction [64]. Selecting an extremely low threshold allows capture of all potential AITD events, but a high false alarm rate can overwhelm clinicians. Conversely, an extremely high decision threshold can greatly reduce the false alarm rate with the cost of failing to detect AITD cases. With an unequal class distribution and high misclassification costs in adverse effect predictions, this study will increase attention paid to future studies to determine the optimal threshold based on the maximum *F*_1_-score with a threshold-moving approach and a KM curve to ensure differentiation ability and clinical justification, rather than using the default cutoff (0.5) directly provided by the machine learning software.

### Clinical Implications

This machine learning model could be used as a clinical decision-making aid for the early and real-time prediction of AITD by incorporating it into computerized physician order entry systems to optimize amiodarone use. This study collected patients’ time-series data to build the model, giving it the capability to provide assessment not only of new amiodarone users but also patients who have used amiodarone for a long time. Patients’ disease status changes over time, so only dynamically analyzing the cumulative dose, duration of therapy, and changes in multiple recent laboratory data will enable simultaneous surveillance. The present model does not provide a one-time glimpse of patient status, but a long-term, real-time prediction. Previous studies that used time-series concepts for intradialytic adverse-event risk prediction also provide evidence for this methodology, as their models had better prediction performance than models lacking features extracted from time-series data [65-67]. Using a threshold-moving system on the PR curve allowed us to further visualize threshold adjustments, balancing high noise and the cost of missing cases for clinical consideration. The model in this study should increase the safety of amiodarone use by enabling individualized risk prediction of AITD.

### Limitations

The study used the Taipei Medical University clinical research database, incorporating data from 3 hospitals in Taiwan. This database provided detailed clinical information, such as laboratory results, cause of death, and time-points for each medical treatment; it has previously been used to successfully predict mortality or classify patients with end-stage liver disease [68]. However, the study was potentially affected by the loss to follow-up of patients, unrecorded disease status, or unrecorded medications due to its nature as a retrospective data analysis. Family history, genetic data, and dietary intake were not documented in the database, but these factors might be integral to the occurrence of AITD. Extrapolation of the study model is thus restricted. Future multicenter, multicountry data are needed to further train and test the model before applying it in a broader clinical setting.

### Conclusions

This study found that XGBoost with the borderline SMOTE resampling technique achieved the best model performance to predict AITD among amiodarone users. Feature selection by RFE and interpretation by SHAP demonstrated good predictive abilities and an explainable model. The optimal point of the threshold was determined to be the one with the maximal *F*_1_-score, found by moving the threshold on the PR curve and differentiating risk groups assessed by the Kaplan-Meier curve. This time-series predictive model can serve as a preliminary tool to support clinicians with individualized AITD risk stratification among amiodarone users.

## Appendix

Multimedia Appendix 1 Definition of preexisting thyroid conditions.

Multimedia Appendix 2 The eligible data collection period and the details of the time-series data collection diagram.

Multimedia Appendix 3 Codebook and missing rate of the features in the study.

Multimedia Appendix 4 Hyperparameters combinations in a grid search and final best hyperparameters.

Multimedia Appendix 5 Pseudocode for grid search, recursive feature elimination (RFE), and five-fold cross-validation.

Multimedia Appendix 6 The formulas of each evaluation metrics and confusion matrix.

Multimedia Appendix 7 The STROBE flowchart of patient selection. WFH: Wan Fang Hospital; TMUH: Taipei Medical University Hospital; SHH: Shuang Ho Hospital; labs: laboratory.

Multimedia Appendix 8 Recursive feature elimination with five-fold cross-validation (RFECV) feature selection.

Multimedia Appendix 9 The training set model performance of accuracy, precision, recall, and F1 score for the raw and resampling methods by 16 machine learning models.

Multimedia Appendix 10 Statistical significances of AUPRCs.

## Abbreviations

AdaBoost: adaptive boosting
AITD: amiodarone-induced thyroid dysfunction
B-SMT: borderline synthetic minority oversampling technique
ENN: edited near neighbors
fT4: free thyroxin
HDL: high-density lipoprotein
ICD: International Classification of Diseases
KM: Kaplan-Meier
KNN: k-nearest neighbor
LDL: low-density lipoprotein
LR: logistic regression
PR: precision-recall
RFE: Recursive feature elimination
RFECV: recursive feature elimination cross-validation
SHAP: Shapley additive explanation
SMOTE: synthetic minority oversampling technique
TSH: thyroid-stimulating hormone
XGBoost: extreme gradient boosting

## Data Availability

The data were obtained from the Taipei Medical University clinical research database; this was approved by the Institutional Review Board of Taipei Medical University (TMU-JIRB-N202107054). Data availability is restricted because of the personal information protection requirement of the institutional review board, but the data are available from the corresponding author on reasonable request.
