# Supplementary material for: Explainable Machine Learning Techniques To Predict Amiodarone-Induced Thyroid Dysfunction Risk: Multicenter, Retrospective Study With External Validation
Source: J Med Internet Res. 2023 Feb 7;25:e43734. doi: 10.2196/43734 (PMC9944157; doi:10.2196/43734)
Supplement: Multimedia Appendix 1 [file jmir_v25i1e43734_app1.docx]

## Multimedia Appendix 1

Multimedia Appendix 1. Definition of preexisting thyroid conditions

| Reason for exclusion | Definitions |
| --- | --- |
| Subclinical thyroid labs | TSH titer >10 mU/l or TSH titer <0.1 mU/l |
| Preexist thyroid disease  (ICD code) | 1. Thyroid carcinoma ICD9: 193,226; ICD10: D34, E31.22 2. Hyperthyroidism Goiter: ICD9: 240, 241; ICD10: E01, E04 Thyrotoxicosis: ICD9: 242; ICD10: E05.0, E05.1, E05.2, E05.3, E05.4, E05.8, E05.9   Thyroiditis: ICD9: 245; ICD10: E06   1. Hypothyroidism Congenital hypothyroidism: ICD9:243; ICD10: E03.0, E03.1   Hypothyroidism: ICD9:244; ICD10: E02, E01.8, E03, E89.0   1. Other thyroid disorder ICD9: 246; ICD10: E07 2. Thyroidectomy ICD9 Procedure Codes: 06.2,06.3,06.4,06.5,06.6,06.98 |
| Thyroid related medications  (ATC code) | Levothyroxine: H03AA02;  Propylthiouracil: H03BA02;  Carbimazole: H03BB01;  Methimazole: H03BB02; |
