# Supplementary material for: Explainable Machine Learning Techniques To Predict Amiodarone-Induced Thyroid Dysfunction Risk: Multicenter, Retrospective Study With External Validation
Source: J Med Internet Res. 2023 Feb 7;25:e43734. doi: 10.2196/43734 (PMC9944157; doi:10.2196/43734)
Supplement: Multimedia Appendix 2 [file jmir_v25i1e43734_app2.docx]

## Multimedia Appendix 2

Multimedia Appendix 2. The eligible data collection period and the details of the time-series data collection diagram.

As shown in Figure S1, up to two years of follow-up of patients was defined in this study. If the patient developed amiodarone-induced thyroid dysfunction (Event) or loss follow-up (Loss follow-up) in the EHR, we stopped the data collection at the previous window to avoid missing thyroid events. The dynamic data collection was performed in the observation periods while the prediction periods were to capture the prediction target (Figure S2). For instance, Patient A developed the event in the 11th month and thus was followed up till the 9th month. Patient B was free from the event and followed till the end of the study, while Patient C lost follow-up at the 14th month, therefore the data collection stopped at the 12th month.


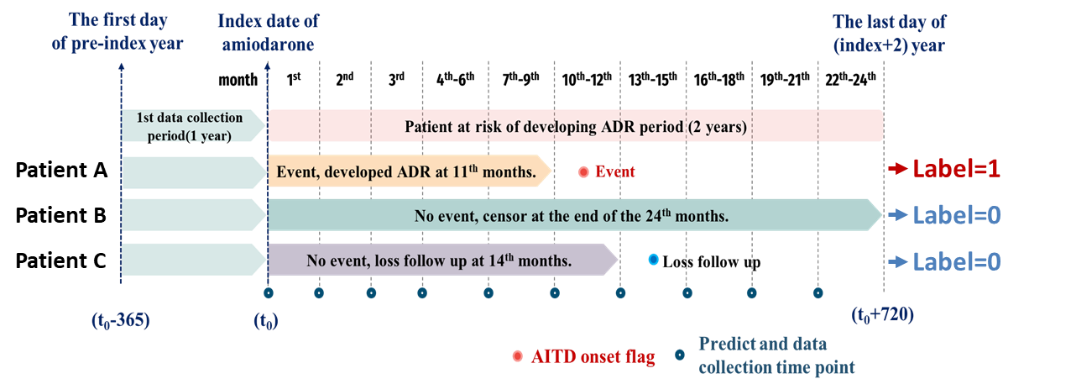
Figure S1. Eligible data collection period of patients


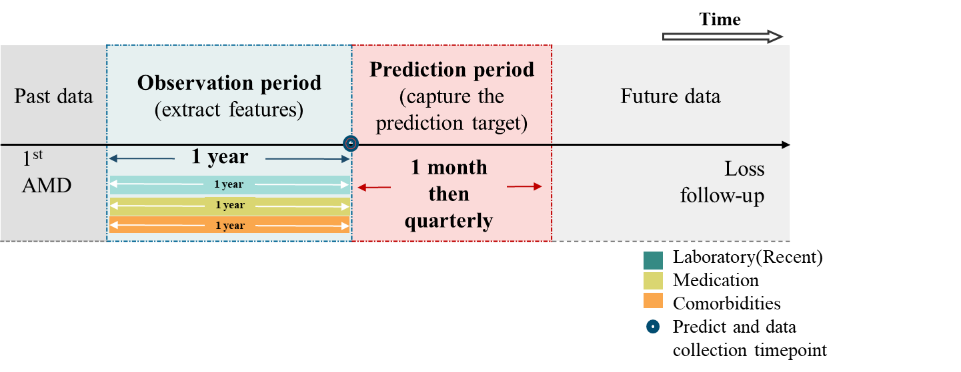

Figure S2. Observation period and prediction period
