# Supplementary material for: Explainable Machine Learning Techniques To Predict Amiodarone-Induced Thyroid Dysfunction Risk: Multicenter, Retrospective Study With External Validation
Source: J Med Internet Res. 2023 Feb 7;25:e43734. doi: 10.2196/43734 (PMC9944157; doi:10.2196/43734)
Supplement: Multimedia Appendix 3 [file jmir_v25i1e43734_app3.docx]

## Multimedia Appendix 3

Multimedia Appendix 3. Codebook and missing rate of the features in the study

| **Variable** | **Full name** | **Type** | **Unit /Assignment** | **Missing rate**  **in the training/test (%)** | |
| --- | --- | --- | --- | --- | --- |
| outcome | --- | Categorical | 0: non-AITD;1: AITD | 0 | 0 |
| Age | Age at index date | Continuous | years old | 0 | 0 |
| Sex | Sex type | Categorical | 0: Female; 1: Male | 0 | 0 |
| Height | Height | Numerical | cm | 8.1 | 16.54 |
| Weight | Weight | Numerical | kg | 9.42 | 15.50 |
| BMI | Body mass index | Numerical | kg/m2 | - | - |
| charlson_TOT_GRP | Charlson Comorbidity Index of last diagnosis [1] | Numerical | the CCI was calculated from the updated CCI version | 0 | 0 |
| Alcohol | Drinking history | Categorical | 0:no,1:yes | 0 | 0 |
| Smoking | Smoking history | Categorical | 0:no,1:yes | 0 | 0 |
| Cumulative_dose_g | The cumulative amiodarone effective dose taken by the patient | Numerical | g | 0 | 0 |
| Aver_dose_kg | $\frac{\text{cumulative dose(g)}}{\text{body weight(kg)}}$ | Numerical | g/kg | 0 | 0 |
| Duration | treatment duration of amiodarone | Numerical | day | 0 | 0 |
| PDD | $\frac{\text{cumulative dose(g)}}{\text{therapeutic days}}$ | Numerical | g/day  The average daily amount of a drug that is actually prescribed. | 0 | 0 |
| Sum days | Therapeutic days (TD) | Numerical | day | 0 | 0 |
| rx_TKIs | prescription of tyrosine kinase inhibitors | Categorical | ATC_CODE: | 0 | 0 |
| rx_allopurinol | prescription of allopurinol | Categorical | ATC_CODE: M04AA01 | 0 | 0 |
| rx_antipsychotic | prescription of antipsychotics | Categorical | ATC_CODE: N05A | 0 | 0 |
| rx_NSAID | prescription of Non-Steroidal Anti-Inflammatory Drugs | Categorical | ATC_CODE:M01A,  except for glucosamine [M01AX05] | 0 | 0 |
| rx_antiDMs | antidiabetics including sulfonylureas, thiazolidinedione (TZD), dipeptidyl peptidase 4 (DPP-4) inhibitors, sodium-glucose cotransporter (SGLT2) inhibitors, and α-glucosidase inhibitors | Categorical | ATC_CODE: A10BB, A10BD, A10BF, A10BG, A10BH, A10BJ, A10BK, A10BK, A10BX | 0 | 0 |
| rx_metformin | prescription of metformin | Categorical | ATC_CODE: A10BD07 | 0 | 0 |
| rx_arrhythmia_type1b | Propafenone | Categorical | ATC_CODE: C01BC03 | 0 | 0 |
| dx_bradycardia | diagnosis of bradycardia | Categorical | ICD 9: 427.81  ICD 10: I49.5, R00.1 | 0 | 0 |
| dx_goiter | diagnosis of goiter | Categorical | ICD9: 240, 241  ICD10: E01, E04 | 0 | 0 |
| dx_anemia | diagnosis of anemia | Categorical | ICD 9: 280, 281, 282, 283, 284, 285  ICD 10: D50, D51, D52, D53,  D55, D57, D58, D59, D60, D61, D63, D64 | 0 | 0 |
| dx_hypertension | diagnosis of hypertension | Categorical | ICD 9: 401, 402, 403, 404, 405  ICD 10: I10, I11, I.12, I13, I14, I15, N26.2 | 0 | 0 |
| dx_diabetes | diagnosis of diabetes | Categorical | ICD 9: 250  ICD 10: E08, E09, E10, E11, E12, E13, E14 | 0 | 0 |
| dx_anemias | diagnosis of anemias | Categorical | ICD 9: 280, 281, 282, 283, 284, 285  ICD 10: D50, D51, D52, D53, D55, D57, D58, D59, D60, D61, D63, D64 | 0 | 0 |
| dx_DMnephropathys | diagnosis of diabetic nephropathy | Categorical | ICD 9: 250.4  ICD10: E10.2, E11.2, E13.2 | 0 | 0 |
| dx_DM_coms | diagnosis of diabetes with complications | Categorical | ICD 9:250.4, 250.5, 250.6, 250.7  ICD10: E08.2, E08.3, E08.4, E08.5, E09.2, E09.3, E09.4, E09.5, E10.2, E10.3, E10.4, E10.5, E10.7, E11.2, E11.3, E11.4, E11.5, E13.2, E13.3, E13.4, E13.5 | 0 | 0 |
| dx_gouts | diagnosis of diabetes with gouts | Categorical | ICD 9:274  ICD 10:M10 | 0 | 0 |
| dx_chronic_RFs | diagnosis of chronic renal failure | Categorical | ICD9:585, 403, 404, V45.1, 996.7  ICD10: N18, T82.4, Z49.2, Z99.2 | 0 | 0 |
| dx_renal_dyss | diagnosis of renal dysfunction [2] | Categorical | ICD 9:  250.3, 250.4, 580.0, 580.9, 580.4, 582.4, 583.4, 599.7, 581.9, 582.0, 582.1, 582.2,582.4, 583.0, 583.1, 583.2,583.6, 583.7, 583.9,585.1, 585.9,590.9, 590.2,588.0, 588.1, 588.8, 588.9, 589.0, 589.1, 589.9, 590.3, 593.0, 593.1, 593.2, 593.6, 753.0, 753.2, 753.4,753.3, 593.9, 586, 587, 403, 404, 250.42, 250.43, 405.01, 405.11, 405.91, 405.02, 405.12, 405.92, 582.89, 580.89, 583.89, 580.81, 581.89, 582.81, 583.81, 590.00, 590.01, 590.80, 590.81, 593.81, 593.82, 593.89, 753.10, 753.11, 753.12, 753.13, 753.14,753.15, 753.16, 753.17, 753.19, 996.73, 996.81, 404.12, 404.13, 404.92, 404.93  ICD 10: N00, N01, N02, N03, N04, N05, N06, N07, N08, N11, N12, N14, N15, N16, N18, N19, N25, N26, N27, N28, I12, I13, Q60, Q61, Q62, Q63, E10.2, E14.2, I15.0, I15.1, N39.1, N39.2, T82.4, T86.1, Z49.0, Z49.1, Z49.2, Z94.0, Z99.2 | 0 | 0 |
| dx_RAs | diagnosis of rheumatoid arthritis | Categorical | ICD 9: 714.0, 714.1, 714.2, 714.3  ICD10: M05, M06 | 0 | 0 |
| lab_TSH | Thyroid Stimulating Hormone | Numerical | μIU/mL | 57.51 | 59.23 |
| lab_FT4 | Free-thyroxine | Numerical | ng/dL | 64.96 | 71.77 |
| lab_rbc | Red blood cells | Numerical | 10^6/uL | 37.63 | 35.96 |
| lab_hgb | Hemoglobin | Numerical | g/L | 33.90 | 30.92 |
| lab_hct | Hematocrit | Numerical | % | 36.88 | 34.96 |
| lab_mcv | Mean Corpuscular Volume | Numerical | fL | 38.02 | 35.96 |
| lab_mch | Mean Corpuscular Hemoglobin | Numerical | pg | 38.13 | 35.96 |
| lab_mchc | Mean Corpuscular Hemoglobin Concentration | Numerical | g/dL | 38.13 | 35.96 |
| lab_tg | Triglyceride | Numerical | mg/dl | 27.59 | 23.85 |
| lab_AST | Aspartate aminotransferase | Numerical | U/L | 38.63 | 29.14 |
| lab_ALT | Alanine aminotransferase | Numerical | U/L | 17.74 | 24.01 |
| lab_HDL | High-density lipoprotein cholesterol | Numerical | mg/dL | 56.67 | 46.99 |
| lab_LDL | Low-density lipoprotein cholesterol | Numerical | mg/dL | 47.75 | 29.31 |
| lab_SCr | Serum creatinine | Numerical | mg/dL | 10.92 | 7.56 |
| lab_ALK_P | Alkaline Phosphatase | Numerical | U/L | 84.26 | 88.98 |
| lab_Cholesterol | Total cholesterol | Numerical | mg/dL | 40.16 | 32.16 |
| TSH_REMARK | The mark of the latest TSH | Categorical | 1: "H","D","R" for high;  -1:"L" for low; 0: Normal | 0 | 0 |
| FT4_REMARK | The mark of the latest FT4 | Categorical | 1: "H","D","R" for high;  -1:"L" for low; 0: Normal | 0 | 0 |
| T3_REMARK | The mark of the latest T3 | Categorical | 1: "H","D","R" for high;  -1:"L" for low; 0: Normal | 0 | 0 |
| SCr_REMARK | The mark of the latest SCr | Categorical | 1: "H","D","R" for high;  -1:"L" for low; 0: Normal | 0 | 0 |
| RBC_REMARK | The mark of the latest RBC | Categorical | 1: "H","D","R" for high;  -1:"L" for low; 0: Normal | 0 | 0 |
| HGB_REMARK | The mark of the latest HGB | Categorical | 1: "H","D","R" for high;  -1:"L" for low; 0: Normal | 0 | 0 |
| HCT_REMARK | The mark of the latest HCT | Categorical | 1: "H","D","R" for high;  -1:"L" for low; 0: Normal | 0 | 0 |
| TG_REMARK | The mark of the latest TG | Categorical | 1: "H","D","R" for high;  -1:"L" for low; 0: Normal | 0 | 0 |
| AST_REMARK | The mark of the latest AST | Categorical | 1: "H","D","R" for high;  -1:"L" for low; 0: Normal | 0 | 0 |
| ALT_REMARK | The mark of the latest ALT | Categorical | 1: "H","D","R" for high;  -1:"L" for low; 0: Normal | 0 | 0 |
| Cholesterol_REMARK | The mark of the latest Cholesterol | Categorical | 1: "H","D","R" for high;  -1:"L" for low; 0: Normal | 0 | 0 |
| HDL_REMARK | The mark of the latest HDL | Categorical | 1: "H","D","R" for high;  -1:"L" for low; 0: Normal | 0 | 0 |
| LDL_REMARK | The mark of the latest LDL | Categorical | 1: "H","D","R" for high;  -1:"L" for low; 0: Normal | 0 | 0 |
| ALK_P_REMARK | The mark of the latest ALK_P | Categorical | 1: "H","D","R" for high;  -1:"L" for low; 0: Normal | 0 | 0 |
| AST_slope  (per year) | the AST change is divided by the interval days by 365 days | Numerical | $\frac{\mathrm{AST}_{-1}-\mathrm{AST}_{-2}}{Day interval}*365$  (In order to normalize inconsistencies in the interval day) | 58.36 | 55.66 |
| ALT_slope  (per year) | the ALT change is divided by the interval days by 365 days | Numerical | $\frac{\mathrm{ALT}_{-1}-\mathrm{ALT}_{-2}}{Day interval}*365$ | 39.56 | 48.39 |
| SCr_slope  (per year) | the SCr change is divided by the interval days by 365 days | Numerical | $\frac{\mathrm{SCr}_{-1}-\mathrm{SCr}_{-2}}{Day interval}*365$ | 29.47 | 26.84 |
| Cholesterol_slope  (per year) | the Cholesterol change divided by the interval days by 365 days | Numerical | $\frac{\mathrm{Cholesterol}_{-1}-\mathrm{Cholesterol}_{-2}}{Day interval}*365$ | 69.13 | 60.57 |

**References**

1. Glasheen, W., et al., Charlson Comorbidity Index: ICD-9 Update and ICD-10 Translation. American health & drug benefits, 2019. 12: p. 188-197. URL: <https://www.ncbi.nlm.nih.gov/pmc/articles/PMC6684052/pdf/ahdb-12-188.pdf> [access: 2022-10-12]
2. Lu, J., et al., Machine learning risk prediction model for acute coronary syndrome and death from use of non-steroidal anti-inflammatory drugs in administrative data. Scientific Reports, 2021. 11(1): p. 18314. [doi: 10.1038/s41598-021-97643-3]
