# Supplementary material for: Explainable Machine Learning Techniques To Predict Amiodarone-Induced Thyroid Dysfunction Risk: Multicenter, Retrospective Study With External Validation
Source: J Med Internet Res. 2023 Feb 7;25:e43734. doi: 10.2196/43734 (PMC9944157; doi:10.2196/43734)
Supplement: Multimedia Appendix 4 [file jmir_v25i1e43734_app4.docx]

## Multimedia Appendix 4 Table S 4.1. Hyperparameters combinations in a grid search of the 16 models

| **Model/Resampling** | **Parameters** | **Values** |
| --- | --- | --- |
| XGBoost | max_depth | [3,5, 8, 10,12,15,18] |
|  | min_child_weight | [0.10, 0.20,0.30,0.40] |
|  | gamma | [0,0.1,0.2,0.4,0.5,0.6,0.7] |
|  | subsample | [i/10.0 for i in range (6,10)] |
|  | colsample_bytree | [i/10.0 for i in range (6,10)] |
|  | n_estimators | [50, 100, 200, 500,1000,1500] |
|  | learning_rate | [0.001, 0.01, 0.1,0.2,0.3] |
| AdaBoost | n_estimators | [10,50,250,1000,1500,2000,  2500,3000] |
|  | learning_rate | [0.1, 0.2, 0.3, 0.4, 0.5, 0.6, 0.7, 0.8, 0.9, 1] |
|  | algorithm | ('SAMME', 'SAMME.R') |
| K Nearest Neighbor | n_neighbors | np.arange(1, 31) |
|  | weights | ['uniform', 'distance'] |
|  | metric | ['euclidean', 'manhattan'] |
| Logistic regression | C | np.logspace(-3,3,7) |
|  | solver | ['lbfgs', 'liblinear', 'sag', 'saga','newton-cg'] |
|  | penalty | ["l1","l2", "elasticnet", "none"] |

Table S 4.2. Final best hyperparameters of each model

| **Models** | **Hyperparameters** | **Value** |
| --- | --- | --- |
| XGB^a^-Raw | gamma | 0.1 |
|  | max_depth | 8 |
|  | min_child_weight | 0.4 |
|  | colsample_bytree | 0.7 |
|  | subsample | 0.8 |
|  | n_estimators | 100 |
|  | learning_rate | 0.2 |
| XGB-B-SMT^b^ | gamma | 0.1 |
|  | max_depth | 10 |
|  | min_child_weight | 0.2 |
|  | colsample_bytree | 0.7 |
|  | subsample | 0.9 |
|  | n_estimators | 1000 |
|  | learning_rate | 0.01 |
| XGB-ENN^c^ | gamma | 0 |
|  | max_depth | 15 |
|  | min_child_weight | 0.2 |
|  | colsample_bytree | 0.7 |
|  | subsample | 0.7 |
|  | n_estimators | 500 |
|  | learning_rate | 0.01 |
| XGB-Hyb^d^ | gamma | 0.6 |
|  | max_depth | 5 |
|  | min_child_weight | 0.1 |
|  | colsample_bytree | 0.6 |
|  | subsample | 0.8 |
|  | n_estimators | 1500 |
|  | learning_rate | 0.1 |
| Ada^e^-Raw | algorithm | 'SAMME.R', |
|  | learning_rate | 0.1 |
|  | n_estimators | 250 |
| Ada-B-SMT | algorithm | 'SAMME', |
|  | learning_rate | 0.3 |
|  | n_estimators | 1500 |
| Ada-ENN | algorithm | 'SAMME', |
|  | learning_rate | 0.6 |
|  | n_estimators | 1000 |
| Ada-Hyb | algorithm | 'SAMME', |
|  | learning_rate | 0.9 |
|  | n_estimators | 250 |
| KNN^f^-Raw | metric | 'manhattan' |
|  | n_neighbors | 30 |
|  | weights | 'distance' |
| KNN-B-SMT | metric | 'manhattan' |
|  | n_neighbors | 30 |
|  | weights | 'distance' |
| KNN-ENN | metric | 'manhattan' |
|  | n_neighbors | 30 |
|  | weights | 'distance' |
| KNN-Hyb | metric | 'manhattan' |
|  | n_neighbors | 29 |
|  | weights | 'distance' |
| LR^g^-Raw  (max_iter=1000) | C | 1 |
|  | penalty | 'l2' |
|  | solver | ‘liblinear' |
| LR-B-SMT  (max_iter=1000) | C | 0.001 |
|  | penalty | ‘none’ |
|  | solver | ‘lbfgs’ |
| LR-ENN  (max_iter=1000) | C | 10 |
|  | penalty | ‘l2’ |
|  | solver | 'liblinear' |
| LR-Hyb | C | 0.1 |
| (max_iter=1000) | penalty | ‘l1’ |
|  | solver | 'liblinear' |

^a^XGB: extreme gradient boosting

^b^B-SMT: Borderline Synthesized Minority Oversampling Technique

^c^ENN: EditedNearestNeighbours

^d^Hyb: hybrid oversampling with Borderline Synthetic Minority Oversampling Technique and undersampling with Edited Nearest Neighbor

^e^Ada: Adaptive Boosting

^f^KNN: K Nearest Neighbor

^g^LR: logistic regression
