# Supplementary material for: Explainable Machine Learning Techniques To Predict Amiodarone-Induced Thyroid Dysfunction Risk: Multicenter, Retrospective Study With External Validation
Source: J Med Internet Res. 2023 Feb 7;25:e43734. doi: 10.2196/43734 (PMC9944157; doi:10.2196/43734)
Supplement: Multimedia Appendix 5 [file jmir_v25i1e43734_app5.docx]

## Multimedia Appendix 5 Multimedia Appendix 5. Pseudocode for grid search, recursive feature elimination (RFE), and five-fold cross validation

| Algorithm step | Pseudocode |
| --- | --- |
| Grid search | 1. Create the machine learning models   For XGBoost, Adaboost, KNN, LogisticRegression:  create_model = XGBoost (initialized parameters)  create_model = Adaboost (initialized parameters)  create_model = KNN (initialized parameters)  create_model = LR (initialized parameters)   1. Create the parameters grid   param_grid = {Put all the interested hyperparameters combination}  X = All the interested features  Y = Prediction outcome  model = XGBoost (put into the create_model)  kfold = StratifiedKFold(Split the data into 5 groups)  grid = GridSearchCV(put into the pre-defined model, param_grid and kfold)  grid_result = grid.fit(X, Y)   1. Calculate the best average precision with the best combination of hyperparameters |
| Recursive feature elimination (RFE) | 1. Create the RFE object and compute a cross-validated score.   model = XGBoost (initialized parameters)  model = Adaboost (initialized parameters)  rfecv = RFECV(StratifiedKFold(Split the data into 5 groups))   1. X = All the interested features   Y = Prediction outcome  rfecv.fit(X, Y)   1. Select the best accuracy collection of features   print("Optimal number of features for model")  print("selected features for model") |
| Five-fold cross-validation | 1. StratifiedKFold(Split the data into 5 groups) 2. Load the optimized model by grid search with the best combination of features by RFE. 3. Each fold would take turns to be the validation fold while the remaining four groups would be the training folds. 4. Calculate the accuracy, precision, recall, F1 score, Area Under the Precision-Recall Curve, and area under the receiver operating characteristic curve of the model. |
