# Supplementary material for: Explainable Machine Learning Techniques To Predict Amiodarone-Induced Thyroid Dysfunction Risk: Multicenter, Retrospective Study With External Validation
Source: J Med Internet Res. 2023 Feb 7;25:e43734. doi: 10.2196/43734 (PMC9944157; doi:10.2196/43734)
Supplement: Multimedia Appendix 6 [file jmir_v25i1e43734_app6.docx]

## Multimedia Appendix 6

Table S 6.1. The formulas of each evaluation metrics

| **Metrics** | **Formula** | |
| --- | --- | --- |
| Accuracy | $\frac{\mathrm{TP}^{a}+\mathrm{TN}^{b}}{TP+\mathrm{FP}^{c}+TN+\mathrm{FN}^{d}}$ | |
| Precision or Positive predictive value (PPV) | $\frac{\mathrm{TP}}{TP+FP}$ | |
| Negative predictive value (NPV) | $\frac{\mathrm{TN}}{TN+FN}$ | |
| Recall (Sensitivity) | $\frac{\mathrm{TP}}{TP+FN}$ | |
| Specificity | $\frac{\mathrm{TN}}{TN+FP}$ | |
| F1 score | | $\frac{2 \times precision \times recall}{precision+recall}$ |
| Geometric Mean  (G-mean) | | $\sqrt{Sensitivity*Specificity}=\sqrt{\frac{TP}{TP+FN}*\frac{TN}{TN+FP}}$ |
| AUROC | | Area Under Receiver Operating Characteristic Curve |
| AUPRC | | Area Under the Precision-Recall Curve |

^a^TP: True positive

^b^TN: True negative

^c^FP: False positive

^d^FN: False negative

Table S 6.2. Confusion matrix

|  |  | Actual values | |
| --- | --- | --- | --- |
|  |  | Positive | Negative |
| Predicted values | Positive | True positive | False positive |
|  | Negative | False negative | True negative |
