# Supplementary material for: Explainable Machine Learning Techniques To Predict Amiodarone-Induced Thyroid Dysfunction Risk: Multicenter, Retrospective Study With External Validation
Source: J Med Internet Res. 2023 Feb 7;25:e43734. doi: 10.2196/43734 (PMC9944157; doi:10.2196/43734)

## Multimedia Appendix 7

Multimedia Appendix 7. The STROBE flowchart of patient selection. WFH: Wan Fang Hospital; TMUH: Taipei Medical University Hospital; SHH: Shuang Ho Hospital; labs: laboratory.


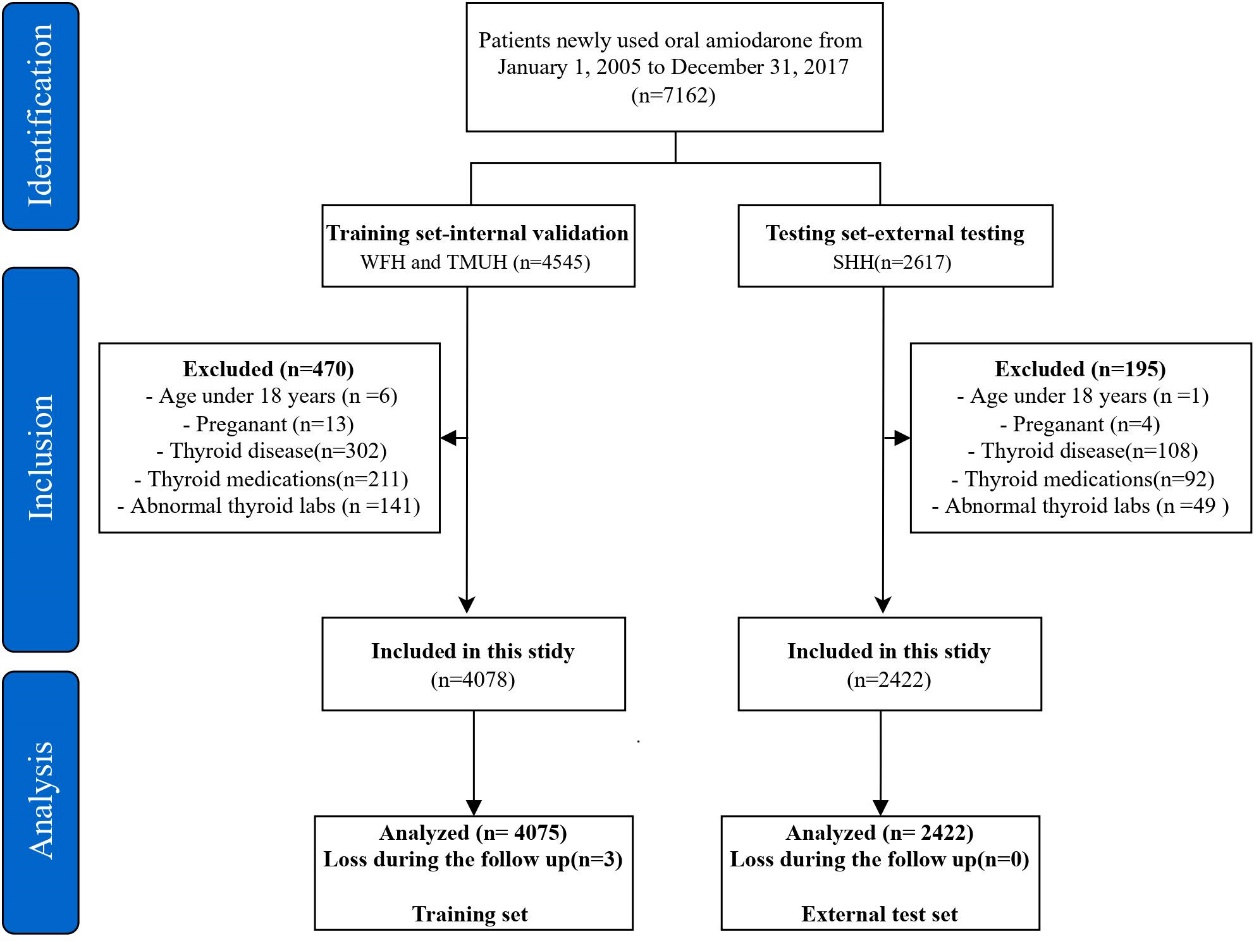

Supplement: Multimedia Appendix 7 [file jmir_v25i1e43734_app7.docx]
