# Supplementary material for: Explainable Machine Learning Techniques To Predict Amiodarone-Induced Thyroid Dysfunction Risk: Multicenter, Retrospective Study With External Validation
Source: J Med Internet Res. 2023 Feb 7;25:e43734. doi: 10.2196/43734 (PMC9944157; doi:10.2196/43734)
Supplement: Multimedia Appendix 8 [file jmir_v25i1e43734_app8.docx]

## Multimedia Appendix 8

Multimedia Appendix 8. Recursive feature elimination with five-fold cross-validation (RFECV) feature selection

Adaboost-RFECV selected 19 features reaching an accuracy of 0.895, while XGBoost-RFECV filtered 46 variables and achieved 0.914(Figure S 8.1). The two feature sets from Adaboost-RFECV and XGBoost-RFECV have the common 19 features (Table S 8.1).

Figure S 8.1. Changes to the model’s performance after applying RFECV based on Accuracy in Adaboost and XGBoost, respectively.


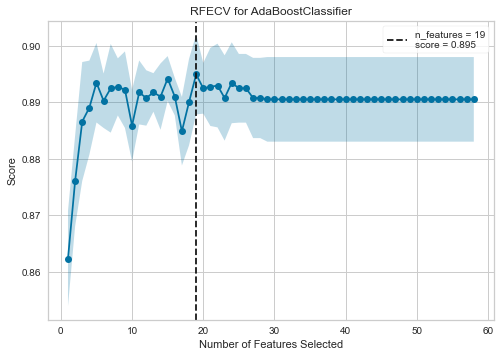

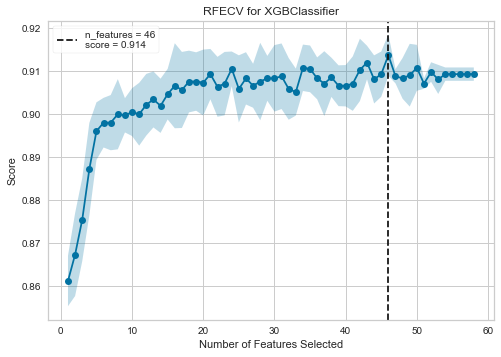


Table S 8.1. Feature selected by Adaboost-RFECV and XGBoost-RFECV

|  | Common selected features by Adaboost-RFECV and  XGBoost-RFECV | Feature selected only by XGB-RFE |
| --- | --- | --- |
| Number of features | 19 | 27 |
| Details of features selected | Cumulative_dose_g, Duration, PDD, AGE, Therapeutic days, BMI, charlson_TOT_GRP, lab_TSH, lab_FT4, lab_ALT, lab_hct, lab_mch, lab_HDL, lab_LDL, lab_ALK_P, lab_Cholesterol, lab_AST, SCr_slope, ALT_slope | SEX_TYPE, Aver_dose_kg,  rx_NSAIDs,rx_allopurinols, rx_antiDMs, rx_metformins, rx_arrhythmia_type1bs,  dx_bradycardias,dx_anemias, dx_hypertensions, dx_diabetess, dx_DMnephropathys, dx_DM_coms, dx_renal_dysfunction,  T3_REMARK, SCr_REMARK, Cholesterol_REMARK, HDL_REMARK, ALK_P_REMARK,  lab_rbc, lab_hgb, lab_mcv,  lab_mchc, lab_SCr, lab_tg,  AST_slope, Cholesterol_slope |
