# Supplementary material for: Explainable Machine Learning Techniques To Predict Amiodarone-Induced Thyroid Dysfunction Risk: Multicenter, Retrospective Study With External Validation
Source: J Med Internet Res. 2023 Feb 7;25:e43734. doi: 10.2196/43734 (PMC9944157; doi:10.2196/43734)
Supplement: Multimedia Appendix 9 [file jmir_v25i1e43734_app9.docx]

## Multimedia Appendix 9

Multimedia Appendix 9. The training set model performance of accuracy, precision, recall, and F1 score for the raw and resampling methods by 16 machine learning models.

| Model | Training set validation fold performance | | | | | |
| --- | --- | --- | --- | --- | --- | --- |
| Accuracy | CV^a^ 1 | CV 2 | CV 3 | CV 4 | CV 5 | Average |
| XGB^b^-Raw | 0.902 | 0.907 | 0.921 | 0.896 | 0.910 | 0.907 |
| XGB-B-SMT^c^ | 0.902 | 0.892 | 0.907 | 0.892 | 0.899 | 0.898 |
| XGB-ENN^d^ | 0.891 | 0.912 | 0.909 | 0.892 | 0.909 | 0.903 |
| XGB-Hyb^e^ | 0.901 | 0.891 | 0.914 | 0.888 | 0.893 | 0.897 |
| Ada^f^-Raw | 0.893 | 0.886 | 0.906 | 0.890 | 0.896 | 0.894 |
| Ada-B-SMT | 0.854 | 0.850 | 0.853 | 0.840 | 0.837 | 0.847 |
| Ada-ENN | 0.876 | 0.887 | 0.901 | 0.859 | 0.890 | 0.882 |
| Ada-Hyb | 0.856 | 0.860 | 0.866 | 0.844 | 0.840 | 0.853 |
| KNN^g^-Raw | 0.872 | 0.865 | 0.865 | 0.865 | 0.87 | 0.867 |
| KNN-B-SMT | 0.62 | 0.621 | 0.687 | 0.634 | 0.64 | 0.640 |
| KNN-ENN | 0.86 | 0.867 | 0.866 | 0.87 | 0.87 | 0.867 |
| KNN-Hyb | 0.629 | 0.667 | 0.699 | 0.650 | 0.667 | 0.663 |
| LR^h^-Raw | 0.858 | 0.861 | 0.854 | 0.851 | 0.849 | 0.855 |
| LR-B-SMT | 0.726 | 0.72 | 0.741 | 0.719 | 0.719 | 0.725 |
| LR-ENN | 0.839 | 0.854 | 0.856 | 0.834 | 0.837 | 0.844 |
| LR-Hyb | 0.744 | 0.736 | 0.757 | 0.735 | 0.736 | 0.742 |
| Precision | CV 1 | CV 2 | CV 3 | CV 4 | CV 5 | Average |
| XGB-Raw | 0.720 | 0.763 | 0.805 | 0.735 | 0.789 | 0.762 |
| XGB-B-SMT | 0.655 | 0.609 | 0.667 | 0.628 | 0.640 | 0.640 |
| XGB-ENN | 0.629 | 0.720 | 0.684 | 0.649 | 0.701 | 0.677 |
| XGB-Hyb | 0.661 | 0.612 | 0.733 | 0.630 | 0.627 | 0.652 |
| Ada-Raw | 0.716 | 0.672 | 0.763 | 0.701 | 0.742 | 0.719 |
| Ada-B-SMT | 0.491 | 0.484 | 0.492 | 0.461 | 0.460 | 0.478 |
| Ada-ENN | 0.561 | 0.592 | 0.645 | 0.508 | 0.606 | 0.583 |
| Ada-Hyb | 0.497 | 0.506 | 0.524 | 0.468 | 0.466 | 0.492 |
| KNN-Raw | 0.929 | 0.875 | 1 | 0.769 | 0.923 | 0.899 |
| KNN-B-SMT | 0.229 | 0.248 | 0.284 | 0.228 | 0.256 | 0.249 |
| KNN-ENN | 0.524 | 0.625 | 0.7 | 0.667 | 0.657 | 0.635 |
| KNN-Hyb | 0.228 | 0.269 | 0.291 | 0.236 | 0.272 | 0.259 |
| LR-Raw | 0.5 | 0.6 | 0.429 | 0.333 | 0.286 | 0.430 |
| LR-B-SMT | 0.307 | 0.3 | 0.325 | 0.28 | 0.308 | 0.304 |
| LR-ENN | 0.403 | 0.479 | 0.5 | 0.339 | 0.389 | 0.422 |
| LR-Hyb | 0.320 | 0.313 | 0.337 | 0.291 | 0.320 | 0.316 |
| Recall | CV 1 | CV 2 | CV 3 | CV 4 | CV 5 | Average |
| XGB-Raw | 0.509 | 0.500 | 0.598 | 0.427 | 0.513 | 0.509 |
| XGB-B-SMT | 0.655 | 0.672 | 0.700 | 0.607 | 0.684 | 0.664 |
| XGB-ENN | 0.569 | 0.621 | 0.684 | 0.538 | 0.641 | 0.611 |
| XGB-Hyb | 0.621 | 0.638 | 0.632 | 0.538 | 0.632 | 0.612 |
| Ada-Raw | 0.414 | 0.388 | 0.496 | 0.402 | 0.419 | 0.424 |
| Ada-B-SMT | 0.733 | 0.784 | 0.769 | 0.650 | 0.795 | 0.746 |
| Ada-ENN | 0.595 | 0.664 | 0.684 | 0.530 | 0.658 | 0.626 |
| Ada-Hyb | 0.724 | 0.750 | 0.752 | 0.632 | 0.769 | 0.726 |
| KNN-Raw | 0.112 | 0.06 | 0.06 | 0.085 | 0.102 | 0.084 |
| KNN-B-SMT | 0.707 | 0.819 | 0.778 | 0.65 | 0.786 | 0.748 |
| KNN-ENN | 0.19 | 0.172 | 0.12 | 0.188 | 0.197 | 0.173 |
| KNN-Hyb | 0.672 | 0.776 | 0.761 | 0.641 | 0.786 | 0.727 |
| LR-Raw | 0.121 | 0.078 | 0.051 | 0.034 | 0.034 | 0.064 |
| LR-B-SMT | 0.733 | 0.724 | 0.744 | 0.607 | 0.769 | 0.715 |
| LR-ENN | 0.267 | 0.302 | 0.23 | 0.162 | 0.239 | 0.240 |
| LR-Hyb | 0.716 | 0.716 | 0.718 | 0.590 | 0.744 | 0.696 |
| F1 score | CV 1 | CV 2 | CV 3 | CV 4 | CV 5 | Average |
| XGB-Raw | 0.596 | 0.604 | 0.686 | 0.541 | 0.622 | 0.614 |
| XGB-B-SMT | 0.655 | 0.639 | 0.683 | 0.617 | 0.661 | 0.651 |
| XGB-ENN | 0.597 | 0.667 | 0.684 | 0.589 | 0.670 | 0.641 |
| XGB-Hyb | 0.640 | 0.624 | 0.679 | 0.581 | 0.630 | 0.631 |
| Ada-Raw | 0.525 | 0.492 | 0.601 | 0.511 | 0.536 | 0.533 |
| Ada-B-SMT | 0.588 | 0.599 | 0.600 | 0.539 | 0.583 | 0.582 |
| Ada-ENN | 0.577 | 0.626 | 0.664 | 0.519 | 0.631 | 0.603 |
| Ada-Hyb | 0.589 | 0.604 | 0.618 | 0.538 | 0.581 | 0.586 |
| KNN-Raw | 0.2 | 0.113 | 0.113 | 0.154 | 0.185 | 0.153 |
| KNN-B-SMT | 0.346 | 0.381 | 0.416 | 0.338 | 0.386 | 0.373 |
| KNN-ENN | 0.278 | 0.27 | 0.204 | 0.293 | 0.303 | 0.270 |
| KNN-Hyb | 0.341 | 0.399 | 0.421 | 0.345 | 0.404 | 0.382 |
| LR-Raw | 0.194 | 0.137 | 0.091 | 0.062 | 0.061 | 0.109 |
| LR-B-SMT | 0.433 | 0.424 | 0.452 | 0.383 | 0.44 | 0.426 |
| LR-ENN | 0.321 | 0.37 | 0.316 | 0.22 | 0.296 | 0.305 |
| LR-Hyb | 0.443 | 0.436 | 0.459 | 0.390 | 0.447 | 0.435 |

^a^CV: cross validation.

^b^XGB: extreme gradient boosting

^c^B-SMT: Borderline Synthesized Minority Oversampling Technique

^d^ENN: EditedNearestNeighbours

^e^Hyb: hybrid oversampling with Borderline Synthetic Minority Oversampling Technique and undersampling with Edited Nearest Neighbor

^f^Ada: Adaptive Boosting

^g^KNN: K Nearest Neighbor

^h^LR: logistic regression
