# Supplementary material for: Explainable Machine Learning Techniques To Predict Amiodarone-Induced Thyroid Dysfunction Risk: Multicenter, Retrospective Study With External Validation
Source: J Med Internet Res. 2023 Feb 7;25:e43734. doi: 10.2196/43734 (PMC9944157; doi:10.2196/43734)
Supplement: Multimedia Appendix 10 [file jmir_v25i1e43734_app10.docx]

## Multimedia Appendix 10.

Multimedia Appendix 10. Statistical significances of AUPRCs^a^

The AUPRC comparison between different resampling methods (Table S10.1) and the best performance XGB-B-SMT compared with other XGBoost-based models (Table S10.2). The 95% confidence interval (CI) differences in areas were calculated by bootstrapping 1000 times[1]. If the 95% CI for the difference in areas does not include 0, it can be refered that these two areas are significantly different (P<.05)

Table S10.1. AUPRCs^a^ comparison between different resampling methods and raw models

| Model | Test set [95% CI] | Difference in areas (95% CI) |
| --- | --- | --- |
| XGB^b^-Raw | 0.742 [0.687~0.790] | Reference |
| XGB-B-SMT^c^ | 0.751 [0.697~0.799] | 0.009579(0.005135-0.01420)^i^ |
| XGB-ENN^d^ | 0.741 [0.686~0.790] | 0.0003068(-0.007749-0.005834) |
| XGB-Hyb^e^ | 0.730 [0.675~0.779] | 0.01156(0.006522-0.01611)^i^ |
| Ada^f^-Raw | 0.643 [0.585~0.697] | Reference |
| Ada-B-SMT | 0.654 [0.596~0.708] | 0.01122(0.001016-0.02328)^i^ |
| Ada-ENN | 0.635 [0.577~0.690] | 0.007929(0.002976-0.01723)^i^ |
| Ada-Hyb | 0.624 [0.565~0.680] | 0.01877(0.009606-0.02613)^i^ |
| KNN^g^-Raw | 0.500 [0.441~0.559] | Reference |
| KNN-B-SMT | 0.470 [0.412~0.529] | 0.02964(0.01380-0.04020)^i^ |
| KNN-ENN | 0.393 [0.337~0.452] | 0.1068(0.09102-0.1224)^i^ |
| KNN-Hyb | 0.467 [0.408~0.526] | 0.03336(0.01648-0.04307)^i^ |
| LR^h^-Raw | 0.294 [0.244~0.351] | Reference |
| LR-B-SMT | 0.303 [0.252~0.360] | 0.008972(0.004832-0.01298)^i^ |
| LR-ENN | 0.300 [0.249~0.357] | 0.005825(0.00003150-0.01186)^i^ |
| LR-Hyb | 0.305 [0.253~0.362] | 0.01049(0.007206-0.01475)^i^ |

^a^AUPRC: area under the precision-recall curve.

^b^XGB: extreme gradient boosting

^c^B-SMT: Borderline Synthesized Minority Oversampling Technique

^d^ENN: EditedNearestNeighbours

^e^Hyb: hybrid oversampling with Borderline Synthetic Minority Oversampling Technique and undersampling with Edited Nearest Neighbor

^f^Ada: Adaptive Boosting

^g^KNN: K Nearest Neighbor

^h^LR: logistic regression

^i^The difference in areas achieved statistical significance (P<.05)

Table S10.2. XGB-^a^ based-model AUPRCs^b^ comparison between resampling methods

|  | XGB-B-SMT^c^,  Difference in areas (95% CI) |
| --- | --- |
| XGB-Raw | 0.009579(0.005135-0.01420) ^f^ |
| XGB-ENN^d^ | 0.02114(0.01464-0.02672) ^f^ |
| XGB-Hyb^e^ | 0.009886(0.0009488-0.01599) ^f^ |

^a^XGB: extreme gradient boosting

^b^AUPRC: area under the precision-recall curve.

^c^B-SMT: Borderline Synthesized Minority Oversampling Technique

^d^ENN: EditedNearestNeighbours

^e^Hyb: hybrid oversampling with Borderline Synthetic Minority Oversampling Technique and undersampling with Edited Nearest Neighbor

^f^The difference in areas achieved statistical significance (P<.05)

**Reference**

1. Boyd, K., K.H. Eng, and C.D. Page. Area under the Precision-Recall Curve: Point Estimates and Confidence Intervals. 2013. Berlin, Heidelberg: Springer Berlin Heidelberg [doi: 10.1007/978-3-642-40994-3_29]
